# High-resolution noise substitution to measure overfitting and validate resolution in 3D structure determination by single particle electron cryomicroscopy^☆^

**DOI:** 10.1016/j.ultramic.2013.06.004

**Published:** 2013-12

**Authors:** Shaoxia Chen, Greg McMullan, Abdul R. Faruqi, Garib N. Murshudov, Judith M. Short, Sjors H.W. Scheres, Richard Henderson

**Affiliations:** MRC Laboratory of Molecular Biology, Francis Crick Avenue, Cambridge CB2 0QH, U.K

**Keywords:** Single particle, Electron cryomicroscopy, Validation, Resolution, Overfitting, Beta-galactosidase

## Abstract

Three-dimensional (3D) structure determination by single particle electron cryomicroscopy (cryoEM) involves the calculation of an initial 3D model, followed by extensive iterative improvement of the orientation determination of the individual particle images and the resulting 3D map. Because there is much more noise than signal at high resolution in the images, this creates the possibility of noise reinforcement in the 3D map, which can give a false impression of the resolution attained. The balance between signal and noise in the final map at its limiting resolution depends on the image processing procedure and is not easily predicted. There is a growing awareness in the cryoEM community of how to avoid such over-fitting and over-estimation of resolution. Equally, there has been a reluctance to use the two principal methods of avoidance because they give lower resolution estimates, which some people believe are too pessimistic. Here we describe a simple test that is compatible with any image processing protocol. The test allows measurement of the amount of signal and the amount of noise from overfitting that is present in the final 3D map. We have applied the method to two different sets of cryoEM images of the enzyme beta-galactosidase using several image processing packages. Our procedure involves substituting the Fourier components of the initial particle image stack beyond a chosen resolution by either the Fourier components from an adjacent area of background, or by simple randomisation of the phases of the particle structure factors. This substituted noise thus has the same spectral power distribution as the original data. Comparison of the Fourier Shell Correlation (FSC) plots from the 3D map obtained using the experimental data with that from the same data with high-resolution noise (HR-noise) substituted allows an unambiguous measurement of the amount of overfitting and an accompanying resolution assessment. A simple formula can be used to calculate an unbiased FSC from the two curves, even when a substantial amount of overfitting is present. The approach is software independent. The user is therefore completely free to use any established method or novel combination of methods, provided the HR-noise test is carried out in parallel. Applying this procedure to cryoEM images of beta-galactosidase shows how overfitting varies greatly depending on the procedure, but in the best case shows no overfitting and a resolution of ~6 Å. (382 words)

## Highlights

• A new method to validate 3D cryoEM maps of biological structures is described.
• High-resolution noise substitution is a tool to measure the amount of overfitting of noise in single particle cryoEM.
• A reliable, unbiased resolution estimation can be obtained even when some overfitting is present.
• Structure of beta-galactosidase at ~6 Å resolution is determined by cryoEM.

## 1 Introduction

Three-dimensional electron cryomicroscopy (cryoEM) of biological assemblies has grown steadily during the last 30 years. The field began with the pioneering work of Dubochet and colleagues, who developed conditions for rapidly freezing thin films of biological macromolecules in amorphous ice [1]. It was helped by the early demonstration that the effect of damage by electron irradiation was less on specimens kept at low temperature [2]. Over the last 25 years, technical advances such as colder and more stable stages [3,4], better vacuum around the specimen with less ice contamination [5], intermediate voltage (300 kV) electron microscopes, high brightness field emission guns [6], improved electron detectors [7], and improved computer programs [8–12] running on much faster computers have all contributed to making it easier to obtain high-resolution structures. There are now many structures at near atomic resolution from two-dimensional (2D) crystals [13–15], helical assemblies [16,17] and icosahedral virus structures with high point group symmetry [18–20], and many lower symmetry single particle structures of smaller molecular mass assemblies at subnanometre resolution [21–24].

As the field has grown, a need for quality control criteria and validation methods has become more important. In a recent report summarising the current consensus [25], it was clear that tools at three levels were required. First, the general correctness of a cryoEM structure, particularly for a new analysis often at low resolution, needs to be proved. Second, we need to be able to show objectively whether resolution estimates are correct. Finally, if an atomic model is fitted into the experimental density map, there must be a way to judge the reliability of the interpretation of density in terms of atoms.

These needs are most vital for single particle cryoEM, because the Fourier transforms of the raw images and the final three-dimensional (3D) map are continuous. They do not have the empty space that is found between the diffraction spots in 2D crystals or between the layer lines for helical assemblies, which can then be used to estimate signal-to-noise ratio. Progress at the first level of overall validation at lower resolution has been made through the use of tilt pair validation [26,27]. For validation of the final interpretation in terms of atoms, the use of Fourier Shell Correlation (FSC) plots between the experimental map and a map calculated from the atomic coordinates that have been used to interpret the map can also be very revealing, provided that excessive flexibility in the modelling is not allowed. Note however that a global FSC plot can never prove whether individual key features are significant or not, if they form only a small part of the overall structure.

In this paper, we focus on the middle level of validation, which concerns the inter-related problems of overfitting and resolution estimation. In conventional single particle cryoEM analysis, the density in a single 3D map is derived from many 2D images of identical structures in different orientations, whose geometrical relationship to the 3D model are deduced by iterative refinement of five parameters (two for position and three for orientation) for each 2D image. If there were accurate prior knowledge of these 5 parameters for each 2D image, the resulting 3D map would consist of a 3D image of the true structure plus random noise arising purely from the statistical nature of the contributing low-dose images. In practice, however, the positions and orientations must be iteratively refined against the gradually improving 3D model. Since the raw images contain much more random noise than real features at high resolution, the positions and orientations are biased slightly by fitting of the 2D noise to features in the 3D map. As the cycles of iteration proceed, some noise features in the 3D map gradually build up and bias the 2D particle parameters so that these false features are locked into the 3D map. The final global convergence of 3D map and 2D parameters thus creates a 3D map that consists of a 3D image of the true structure, plus genuine random noise, plus a third component that consists of noise arising from overfitted parameters. In this paper, we use the terms overfitted noise, overfitted parameters and overfitting in general to describe this phenomenon. The term over-fitting was first used in a similar context by Stewart and Grigorieff [28]. Mild overfitting produces additional features in the 3D map with a Fourier power that is less than that arising from the purely random component in the original 2D images. Significant overfitting is where the Fourier power due to overfitting exceeds that expected from noise in the original 2D images. In this paper, we apply a number of different computational procedures to produce 3D maps that display different degrees of overfitting and show how the degree of overfitting can be measured and the resolution reliably estimated in each case.

The problems of both overfitting and resolution estimation have been the subject of a great deal of discussion over the last few years, so it is useful to review what has been said before. We then describe our proposed tool to allow definitive measurement of any overfitting, however large or small, that is associated with the processing of a particular data set, and to validate the estimate of the overall global resolution of a 3D map. We deliberately aimed to develop a procedure that avoided using atomic coordinates for validation, since once a structure reaches the stage of being interpretable in terms of a model, such as when α-helices or β-sheets are recognisable, the FSC between map and model is a reliable way to evaluate both the procedure used and the final map.

Our proposal is to process, alongside the original data set, a data set in which the raw images are modified by high resolution noise (HR-noise) substitution, and to compare the resulting FSC curves. This procedure can be used for validation whether or not an atomic interpretation is proposed or available. The HR-noise substitution procedure may also be useful to measure undesirable resolution artefacts due to masking that may affect the FSC. The procedure we describe does not test the correctness of any low-resolution starting model: another procedure such as tilt pair validation is needed for that. Nor does it test whether the final map retains any initial model bias.

### 1.1 Background and history of overfitting and resolution estimation

The Fourier transforms of single particles are continuous, so it is not as easy to determine signal-to-noise ratio as a function of resolution as it is with 2D crystals [29] or helices [30] where the Fourier transforms are geometrically sampled in a way that allows local estimation of background noise.

The early introduction of the Fourier Shell Correlation (FSC) function [31] and its 2D equivalent the Fourier Ring Correlation [32,33] provided a single particle counterpart to signal-to-noise ratio estimation. In this procedure the data is normally divided into two half sets, which can then be compared for consistency as the structure analysis proceeds. The cross-correlation coefficient, abbreviated to cross-correlation (CC), between 3D maps calculated from the two half data sets is plotted in resolution shells and indicates where the observations are in good agreement (CC=1.0) or where the data are essentially uncorrelated and consist only of noise (CC=0.0). Other resolution-dependent criteria, such as differential phase residual (DPR) or spectral signal-to noise ratio (SSNR), are related ways of comparing and displaying the same information (reviewed in [34, 35]). After obtaining an initial rough 3D structure by using one of a number of possible procedures, such as random conical tilt [36] or angular reconstitution [37], the initial map is used as a 3D reference to refine the single particle orientation parameters. The structure can then be iteratively improved, alternating position and orientation determination with 3D reconstruction. The FSC or other resolution criteria can be used to follow the progress of the structure refinement, which is terminated when there is no further improvement.

These procedures and the accompanying resolution criteria worked adequately for structures calculated from images of negatively stained particles, where signal-to-noise ratio is high. However, problems appeared when 3D structures were calculated from cryoEM images of ice-embedded single particles, caused by the lower contrast and higher noise, resulting in the need to combine many more single particle images. One of the first papers to note the new problems posed by the intrinsically lower signal-to-noise ratio [38] in cryoEM images showed a simulation in which 128 images of pure noise produced a striking resemblance to a reference image consisting of six discs used for alignment by cross-correlations methods, simply by reinforcing the components in the noise image that agreed with those in the reference. Similar simple illustrations of how model bias can be produced by overfitting noise have been taught in cryoEM courses for many years. A popular demonstration is to show how easily a photograph of a well-known person, such as Albert Einstein, can be extracted from relatively small numbers of pure noise images [39].

Grigorieff showed [40] that when signal-to-noise ratio in the images becomes low enough, it is impossible to avoid overfitting when the two half sets are aligned against the same reference structure, regardless of how the procedure is initiated. He was the first to conclude that a reliable estimation of resolution using FSC can be obtained only when the two half data sets are independently aligned against two independent reference structures. In addition, it is well known that masking of 3D maps can introduce false apparent FSCs at high resolution [35]. We also note that, if the two 3D structures have no symmetry, they have to be aligned correctly in position and orientation before the FSC comparison, and even these six parameters can introduce a small degree of apparent correlation if the structure is at very low resolution. Finally, the two independent halves of the data still each have their own independent but uncorrelated degree of overfitting, and this can give rise to some overfitted power in the final averaged 3D map at high resolution. However, at least the resolution estimate is honest and the FSC curve can be used to deduce figure-of-merit (FOM) weights to damp the overfitting at high resolution, as proposed [27]. A number of interesting but pathological FSC curves, which illustrate some of the above points, are shown in Penczek's review on resolution [35].

There have been a number of attempts to improve the accuracy of alignment of the individual images against the 3D model during the iterative refinement procedure, and to minimise the build-up of overfitted noise. For example, Stewart and Grigorieff [28] introduced a reciprocal-space weighting scheme. This scheme maximised a resolution-dependent function, proportional to CC^3^, in Frealign so that the alignment would be guided only by regions of the molecular transform where diffraction was strong and would ignore regions where the noise was dominant. In addition to using this weighting scheme, they also maximised the modulus of CC rather than CC itself, so that some of the very noisy images with large negative values of CC would be subtracted from the 3D average and so counteract the noise accumulation. These two improvements, implemented in Frealign [9] greatly reduced the tendency to the reinforcement of noise in the iteratively refined 3D maps, but did not eliminate it. Scheres introduced a Bayesian interpretation of the cryoEM method that led to an optimal 3D linear filter [41], which required none of the arbitrary parameter tuning that had characterised many earlier methods. The only adjustable parameter in the original work [41], T that was tuned to 4, was made unnecessary by subsequent introduction of an FSC-based variant of the method [10].

In EMAN2 [11], where images are matched against projections of the 3D reference, the particles that end up in each projection-matched bin are then subjected to iterative alignment and exclusion of outlying particles as part of class-averaging. This extra step has the effect of partially decoupling the new 3D map from the 3D map in the previous refinement cycle and has helped to make EMAN2 a popular, robust and efficient 3D reconstruction package, which is generally agreed to do a reliable job of getting from a set of raw particle images to an initial 3D map. However, in general, when a single 3D reference is used to refine the orientations of all the images at each step, overfitting is often present.

In other words, although it has been known for at least 12 years that overfitting, with its accompanying overestimation of resolution, can be avoided by the strict quarantine of the two half data sets throughout the refinement procedure, this separation has not been widely adopted. It is still common practice to carry out the iterative refinement against a common 3D reference and then, at the very end, to split the data set into two halves and calculate the FSC, which as a result cannot be used to distinguish genuine structure from overfitted noise. The problems of reliable resolution estimation and overfitting of noise have therefore become intertwined.

It has also been shown [9,27] that perfectly good orientations can be obtained when the resolution used in orientation determination is restricted to a zone that excludes very weak high-resolution data. In the cryoEM analysis of pyruvate dehydrogenase [27], the use of data to a resolution of 15 Å for orientation determination allowed a 3D map with 8.7 Å resolution to be obtained. Because the data beyond 15 Å were never used in the refinement, there was no possibility of overfitting and the 8.7 Å resolution achieved was unbiased. It was also shown [27] that an FSC of 0.143 that relates the two half sets in such an unbiased refinement is equivalent to an FSC of 0.5 between the structure calculated from all the data and a perfect reference structure if it could be obtained.

A variant on the exclusion of the highest resolution shells of reciprocal space during particle alignment was suggested by Shaikh et al. [42]. They proposed the exclusion of a shell of, for example, medium resolution data, so that any positive value of the FSC in that shell could be used to cross-validate the 3D model. They also demonstrated, using images consisting entirely of pure noise, that overfitting was easily introduced but that information not used in refinement cannot be overfitted. However, the exclusion of a medium or low-resolution shell is less desirable than the exclusion of the outermost shell where the structure factors are weakest because these medium and low resolution Fourier components are most important in alignment.

The use of these above two procedures for alignment, namely the splitting of data into two completely independent halves, or exclusion of the highest resolution (and weakest) zone have thus been known for many years to allow structure analysis that avoids overfitting and gives reliable resolution estimates. However, neither has been popular for two reasons, one logical and one cosmetic. The logical reason is that the exclusion of any data that could help orientation determination must reduce the accuracy of a structure determination. The two independent 3D maps, each calculated from half the data, must be less accurate, and therefore the particle orientations will be less accurate and therefore the final structure calculated by averaging the two maps will be less accurate. The cosmetic reason is that any overfitting that cannot be distinguished from a genuinely higher resolution makes the results of a research project appear better than they are, thus (falsely) impressing colleagues, referees and grant-awarding bodies.

A good understanding of how this situation arose is apparent from a recent publication that discusses the definition and estimation of resolution in single particle reconstructions [34]. In this paper, the authors explain well the different resolution criteria that have been developed and how noise bias and model bias can be introduced by the different 3D reconstruction procedures. They acknowledge that special care must be taken to ensure statistical independence of the two half-sets and that both reference-based and reference-free alignment inevitably introduce statistical dependencies and cause a resolution overestimation. They are also concerned that splitting the data into two halves results both in poorer statistics for each map and a more pessimistic estimate of resolution since the two maps contain only half the data and hence less information. The implication is that the effects of overfitting (increasing the apparent resolution) are counterbalanced by the poorer statistics of the half data sets (decreasing resolution). They also observe that an FSC of 0.5 is the resolution criterion encountered most often and note that the two shortcomings bias this resolution estimation in opposite directions. In a recent review, Grigorieff and Harrison [43] also state explicitly that, in cases where reconstructions are affected by overfitting of noise, it may be necessary to compensate by reporting the resolution at FSC 0.5.

The main argument for using all the images to calculate the best 3D map at each cycle and using all the diffraction data out to the highest possible resolution for the orientation determination is that the orientations obtained must be more accurate if the 3D reference contains all the information available. So it is understandable that there has been a reluctance to split the data into two halves and keep them completely separate, or to impose a resolution cut-off in data used for orientation determination. Both these procedures, in different ways, use less than the complete available information for parameter refinement.

The Protein Data Bank organised a meeting of an electron microscopy validation task force (EMVTF) around these topics to discuss the future development of validation criteria and try to extract some kind of consensus [25]. Participants at the meeting agreed that the field had not yet developed rigorous methods to ensure the correctness or reliability of electron microscopy structures, but felt that their development was a high priority and made a number of recommendations. In one part of the discussion, specifically focussed on single particle cryoEM, the term “gold-standard FSC” was introduced to denote the calculation of the FSC between two completely independent halves of a data set that had been separated at the start of a 3D structure analysis and compared only after the refinement had been completed. Subsequently, this was built on by Scheres and Chen [44], who referred to the FSC calculated between two halves of data refined iteratively against a common 3D reference as “sub-optimal FSC” and that between fully independent halves refined iteratively against separate 3D references as “gold-standard FSC”, and showed that use of the gold-standard approach did not yield lower resolution. The work described here represents an attempt to develop an objective and reliable method for estimation of resolution and overfitting that can be used in any image processing protocol.

Many image processing packages, as an integral part of the process, subject the 3D map at each cycle to a low pass filter that removes the high-resolution data that is most susceptible to overfitting before it is used as a 3D reference for the next cycle of orientation determination, sometimes increasing the resolution as the refinement proceeds [45–47]. In some methods, a low-pass resolution limit is imposed automatically in a self-adaptive manner [48]. The application of a low pass filter at each cycle is effective but the nature of the low pass filter may be rather arbitrary and not well-matched to the resolution at which signal-to-noise ratio becomes poor, especially when that resolution is unknown in advance. One idea has been to use the FSC curve at the end of each cycle as the low-pass filter function. However, if overfitted noise builds up in the FSC, then the low pass filter will be more biased to higher resolution than it should be and will lead to more overfitting, and more importantly probably also worse orientations and a worse structure.

In a recent advance, Scheres and Chen [44] introduced a valuable variant on this use of the gold-standard FSC, which can be implemented as part of any image-processing package. The data set is randomly split into two completely separate half sets but the gold-standard FSC, calculated after each cycle, is then used as the low pass filter to provide the weights. In this way, an unbiased and true estimate of signal-to-noise ratio in the images is obtained and then used to extract the right amount of information at different resolutions to optimise the orientation determination. In this case, as well as in any other gold-standard protocol, there is still a small amount of overfitting in each of the separate halves but it does not show up in the gold-standard FSC, which is completely objective. We will demonstrate later in this paper that this procedure also avoids any overfitting in our HR-noise test. Also, since it uses optimal gold-standard FSC weighting, it should give better orientations, with a final 3D map that appears to be as good as or sometimes better than a map calculated using sub-optimal FSC [44]. However, there is still a concern that the accuracy of the orientations might be higher if the 3D reference map was calculated with all the data rather than half of it. This also remains a concern if the orientation refinement is carried out with a strict high-resolution cut-off.

### 1.2 Concept

The core idea we propose to get round all these problems is therefore to create a second stack of single particle images that are identical in every way to the original raw particle images, except that the structure factors (i.e. the individual Fourier components represented by the amplitude and phase at each reciprocal space pixel) beyond a certain chosen resolution are substituted by pure high-resolution noise (HR-noise). This second data set is then subjected to the identical image processing procedure used for the original experimental data, followed by calculation and comparison of the FSC between the two halves of the data. We refer to the two FSC curves as FSC_t_ when carried out with the original data set and FSC_n_ for the HR-noise data set. Any non-zero FSC_n_ values beyond the resolution where the HR-noise was introduced is then due to overfitting and the true resolution obtained can be determined by a comparison of FSC_t_ with FSC_n_. Since the experimenter is allowed to apply any 3D reconstruction method or combination of methods, including new algorithms or tricks of their choice, we hope it will become a popular as well as foolproof method of measuring overfitting and validating resolution. Since the HR-noise data set contains very slightly less information about the structure than does the original data set, it is possible that the particle orientations obtained may be very slightly less accurate, but this should not affect the value of FSC_n_ at high resolution, where only noise is being fitted. We estimate, using Scheres' estimate of the contribution of different frequencies to orientability [10], that HR-noise substitution for the images in this work reduces the information used for orientation determination by only a few per cent.

The build-up of noise after many cycles of 3D reconstruction and orientation refinement depends on many parameters including the detector resolution and particle masking, both of which affect inter-pixel correlation. Therefore, to ensure that the substituted HR-noise data set responds in the same way to the 3D reconstruction procedure as does the original data, the high frequency noise should have the same power spectrum as the raw data, and preferably also the same inter-pixel correlation. The raw image data can come from detectors with different point spread functions and correspondingly different modulation transfer functions and from specimens prepared in different ways sometimes on a carbon substrate with an underlying non-zero noise contribution. Consequently, the simplest way to obtain noise with exactly the same power spectrum and inter-pixel reciprocal-space correlation as found in the particles is to use an adjacent featureless area of the image close to each particle. Since picking the noise regions can be tedious and may be a barrier to its ease of use, we have compared the substitution of high-resolution noise from an area in each micrograph that is adjacent to each particle with the simpler procedure of randomising the phases beyond the chosen resolution. Since we find no difference in outcome between these two methods of introduction of HR-noise, we recommend high-resolution phase randomisation as the preferred procedure in practice. We hope this will be useful to evaluate established procedures as well as future innovations.

## 2 Materials and methods

Specimens were prepared by application of 3 μl drops of a 1 mg/ml solution of *E.coli* beta-galactosidase (Sigma, G3153) in 30 mM phosphate buffer at pH 7.0 to Quantifoil grids (1.2 μm holes), followed by blotting for 5 s and plunge freezing into liquid ethane at near liquid nitrogen temperature. Grids were transferred into an FEI Polara G2 electron microscope and low-dose images recorded either on film at 80 keV and 39,000x magnification or on a Falcon II CMOS direct electron detector at 300 keV and 80,240x magnification (59,000x nominal). The pixel size was 6 μm on film and 14 μm on the Falcon detector, which translates into a sampling rate of ~1.5 Å/pixel with film and ~1.75 Å/pixel with the Falcon. Both nominal pixel sizes required later refinement by up to 3% against an atomic model from X-ray crystallography [49] to obtain the correct magnification.

Two complete data sets were collected. The first consisted of 52 images on film. The second consisted of 89 images on the FEI Falcon II CMOS detector. Stacks of particles were picked by hand using Ximdisp [50], CTF estimated using CTFFIND3 [51], boxed particle densities floated and normalised using BOXIMAGE [52], and 3D maps calculated using Frealign [9], Relion [10], Xmipp [53,54] and a new program still under development by McMullan (unpublished).

A starting reference map was calculated from the atomic coordinates of an *E. coli* beta-galactosidase structure [49], using PDB coordinates 3I3E. A rough correction for the fact that the real specimen is embedded in a solvent (amorphous ice) with a density of ~80% of that of protein was applied by subtracting from a map calculated from atomic coordinates *in vacuo*, another map with a B-factor of 2000 and a scale factor of 0.8. This solvent correction affects only the low-resolution structure: its effect falls to about a third at 20 Å and is negligible by 10 Å. The 80% factor is a rough approximation for the amount of electron scattering by ice compared with protein although a slightly lower value of 72% was estimated previously [27]. Note that we could equally well have used a good experimental cryoEM map as the starting reference, e.g. from reference [26]. However, since we are addressing the issue of overfitting that arises from build-up of noise after many cycles of iterative refinement, the nature of the initial model is not important as long as it provides a reliable first cycle. We do not address the separate problem of initial model bias.

The high-resolution noise substitution was performed by a programme named *makestack_HRnoise*, which substitutes either the amplitudes and phases from an accompanying stack of featureless areas selected from adjacent regions of the same images, or alternatively randomises the phases of the particle structure factors beyond a chosen resolution. The first option creates high-resolution noise with the same image statistics including adjacent inter-pixel correlation. The second option retains the original particle amplitudes but removes any phase correlations between adjacent pixels. Exploring both types of noise substitution was designed to ensure that the HR-noise test represented an information-free control.

Image processing of the particle stacks to produce 3D maps was carried out using the standard single particle iterative procedure of parameter determination and 3D structure calculation, following the normal protocols in each case, as for example with Frealign [9], Relion [10] or Xmipp [53]. We named the FSC curves obtained using the original stack of particles FSC_t_, and the FSC for the HR-noise substituted stack FSC_n_. If we assume that the amount of overfitted noise in FSC_t_ is similar to the amount in FSC_n_ and that the difference represents the true correlation of genuine structural features, we can deduce the value of FSC_true_ as follows.

If *S* represents a Fourier component of the structure, *N*_of_ represents overfitted noise and *N*_ran_ represents random noise, then the signal-to-noise ratio (SNR) and FSC are given by

$${\mathrm{SNR}}_{t}=\Sigma [S^{2}+{N_{\mathrm{of}}}^{2}]/\Sigma [{N_{\mathrm{ran}}}^{2}]\;={\mathrm{FSC}}_{t}/[1-{\mathrm{FSC}}_{t}]$$

$${\mathrm{SNR}}_{n}=\Sigma [{N_{\mathrm{of}}}^{2}]/\Sigma [{N_{\mathrm{ran}}}^{2}]={\mathrm{FSC}}_{n}/[1-{\mathrm{FSC}}_{n}]$$

$${\mathrm{SNR}}_{\mathrm{true}}=\Sigma [S^{2}]/\Sigma [{N_{\mathrm{of}}}^{2}+{N_{\mathrm{ran}}}^{2}]\;\;={\mathrm{FSC}}_{\mathrm{true}}/[1-{\mathrm{FSC}}_{\mathrm{true}}]$$

Note that only random noise appears in the denominator of SNR_t_ and SNR_n_, but both overfitted and random noise appear in the denominator of SNR_true_.

(1) $${\mathrm{FSC}}_{t}=\Sigma [S^{2}+{N_{\mathrm{of}}}^{2}]/\Sigma [S^{2}+{N_{\mathrm{of}}}^{2}+{N_{\mathrm{ran}}}^{2}]$$

(2) $${\mathrm{FSC}}_{n}=\Sigma [{N_{\mathrm{of}}}^{2}]/\Sigma [{N_{\mathrm{of}}}^{2}+{N_{\mathrm{ran}}}^{2}]$$

(3) $${\mathrm{FSC}}_{\mathrm{true}}=\Sigma [S^{2}]/\Sigma [S^{2}+{N_{\mathrm{of}}}^{2}+{N_{\mathrm{ran}}}^{2}]$$

It is easiest to prove the following equation is true by substituting (1) and (2) into Eq. (4) to derive (3).

(4) $${\mathrm{FSC}}_{\mathrm{true}}=({\mathrm{FSC}}_{t}–{\mathrm{FSC}}_{n})/(1–{\mathrm{FSC}}_{n})$$

Eq. (4) thus allows an estimate to be made of the true FSC for the structural features in the map by redefining the overfitted features as noise rather than signal. The formula applies only at resolutions above which HR-noise was substituted. Examples of the application of this formula are shown later in Fig. 3(a) where there is a small amount of overfitting, in Fig. 3(d) where there is a much larger amount, and in Fig. 5(d) where there is an intermediate amount. In each example, FSC_true_ has been calculated from FSC_t_ and FSC_n_.

## 3 Results

**Fig. 1:**
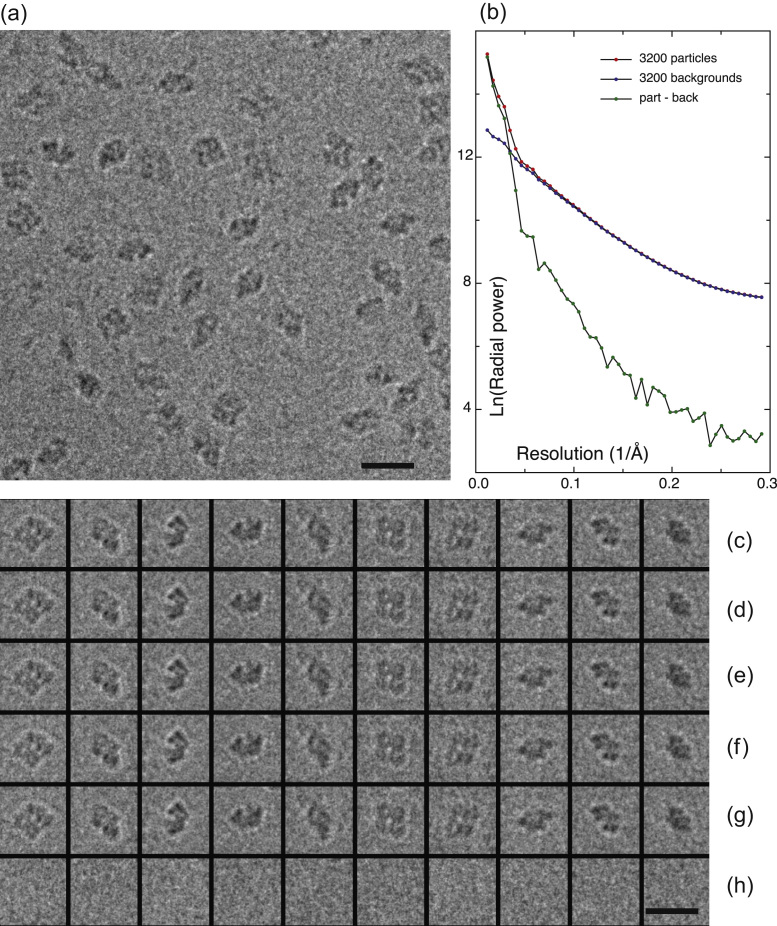
(a) Part of micrograph 01.49.47 recorded at 300 keV on a Falcon II detector showing a field of view of beta-galactosidase particles embedded in ice, with 2.7 μm defocus, (b) average radial power spectra (intensities) of 3200 tightly masked (170×170 Å^2^ box) particle images from 5 micrographs similar to that shown in (a) compared with the same number of background regions. The lower line shows the power in the particles after subtraction of background. Signal is about equal to background at 30 Å resolution, about 40x less than background at 10 Å and 100x less than background at 5 Å resolution. (c) 10 individual particles selected from the micrograph, (d) same particles after HR-noise substitution beyond 10 Å from the adjacent empty areas shown in bottom row, (e) with random phases beyond 10 Å, (f) with HR-noise beyond 17 Å, (g) with random phases beyond 17 Å, and (h) adjacent noise areas used for HR-noise substitution. Since the signal is less or much less than the noise at 17 or 10 Å resolution, the particle images look very similar by eye. Scale bars 200 Å.

A small region of a typical image of beta-galactosidase is shown in Fig. 1(a). Some picked particles are shown in Fig. 1(c), and adjacent featureless areas in Fig. 1(h). The same particles after substitution with HR-noise at two different resolutions are shown in Fig. 1(d)–(g). We were also interested to know how the radial power spectrum of the particle regions differed from that of the adjacent featureless regions. This is shown in Fig. 1(b), which compares the averaged radial power spectra of 3200 particles with that of 3200 background regions from 5 micrographs. The greater power in the particle transforms is clearly visible out to ~10 Å resolution, but beyond that the two curves get closer. Nevertheless, the radial power spectrum from the particles is always higher than for the same amount of background, dropping to around 1% at 3–4 Å. If the same data are plotted on a linear scale, then the Thon rings from the particle transforms are clearly visible (data not shown). However, there are also weaker Thon rings present in the radial power spectrum from the background areas. We think this is due to fragments of degraded protein or impurities, since any Thon rings from pure ice should be too weak to be observed. The difference in diffraction between particles and background extends to high resolution and presumably represents the power of Fourier components of the particle structure, most of which should be due to reproducible features of the 3D structure that the single particle image processing procedure aims to extract. However, the two curves are extremely close, especially at the higher resolutions where HR-noise is being substituted.

**Fig. 2:**
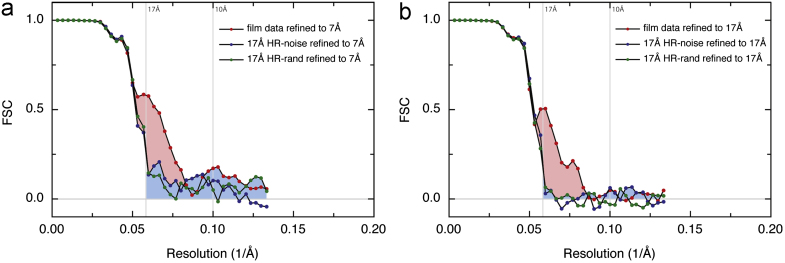
(a) and (b) Results of Frealign processing of 6733 single particle images of beta-galactosidase recorded at 80 keV on film. The FSC between half data sets (red symbols) is compared with that obtained from the same data set with HR-noise substituted beyond 17 Å (blue and green symbols). Overfitting is shaded blue, with the difference between the two curves, representing real features of the structure, shaded pink. (a) FSCs between half data sets with position and orientation refinement to 7 Å resolution. (b) For the same 6733 particles, FSCs between half data sets with position and orientation refinement to 17 Å resolution. No overfitting is present beyond 17 Å in (b) because that information was not used in refinement. Regardless of the cut-off resolution used in refinement, the plots show the images contain structural information to about 13 Å resolution. (For interpretation of the references to colour in this figure legend, the reader is referred to the web version of this article.)

The particle images recorded on film were processed using Frealign and the results are shown in Fig. 2(a) and (b). If the orientation parameters are refined using information that extends to high resolution, in this case ~7 Å, then overfitting is evident in the HR-noise substituted map beyond the 17 Å resolution at which the Fourier components were replaced by noise from the adjacent regions. The blue shading shows the overfitted part of the FSC curve and the difference between FSC_t_ and FSC_n_, shaded in pink, shows the amount of true signal from real features of the structure. It is clear that, from 6733 particle images on film at 80 keV, the resolution reaches about 13 Å. If the orientation parameters are refined using only data to 17 Å, Fig. 2(b) shows that the structure is just as well-determined as shown by the region of pink shading, but there is then no overfitting, as expected. If the two maps produced by refining orientations to 17 Å or 7 Å are then compared with a map from atomic coordinates, then virtually superimposable FSC curves (between map and model) are obtained (data not shown). The image processing thus behaves as expected; refining orientations only at medium resolution completely avoids overfitting at high resolution, whereas refining orientations at high resolution does produce some overfitting. This can now be measured quantitatively as shown in Fig. 2(a). We then repeated the calculations, using HR-noise substituted particles where the phases of the particle structure factors were simply randomised (rather than replacing both amplitudes and phases with those from adjacent featureless regions) and obtained very similar results (green symbols in Fig. 2(a) and (b)). Note that, due to the limited number of particles, the error level in the FSC is about +/−0.05, so any non-zero values beyond about 13 Å resolution in this case represent overfitting. We therefore recommend the simpler introduction of HR-noise by phase randomisation. Applying the same procedure to a larger number of particles (50330) from 52 film images improved the true resolution to ~11 Å and showed a similar amount of overfitting if the orientation refinement included high resolution data (data not shown).

**Fig. 3:**
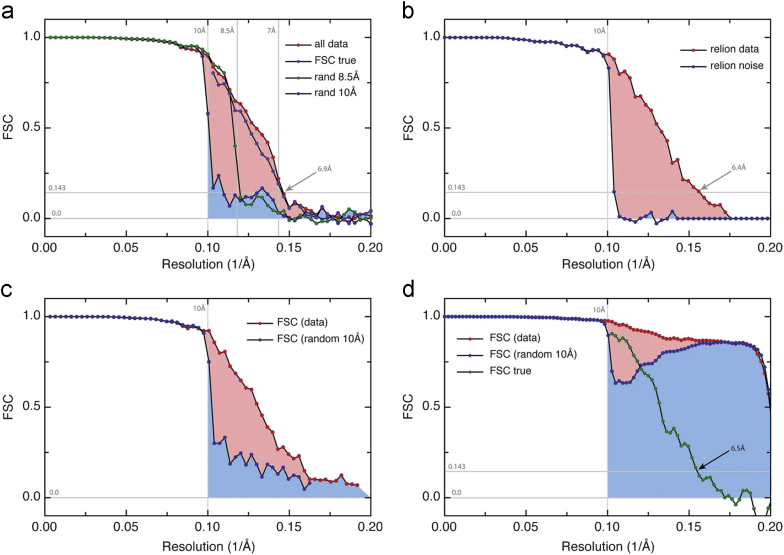
Results of processing of 43758 single particle images of beta-galactosidase recorded at 300 keV on the FEI Falcon II detector, using four different procedures. The FSC from the particle data set (red symbols) is compared in each case with that obtained from the same data set with HR-noise substituted beyond 10 Å (blue symbols) or in one case beyond 8.5 Å. Overfitting is shaded blue, with the difference between the two curves, representing correlations between real features of the structure, shaded pink. (a) Results of Frealign processing. To produce more noticeable overfitting, a value of −200 Å^2^ for the parameter RB-factor was used, noting that this is explicitly not recommended for normal practice [9]. Data out to 7 Å resolution was used in orientation determination, so the overfitting is only evident between 10 and 7 Å resolution. Calculation of FSC_true_ from FSC_t_ and FSC_n_ demonstrates a resolution around 6.9 Å. In this case, the very small degree of overfitting does not affect the estimated resolution. (b) The Relion package has been used with complete separation of the data into two halves and gold-standard FSC weighting to carry out low-pass filtering of the reference at each cycle as described [44]. With the gold-standard procedure and gold-standard FSC weighting, there is no overfitting, confirmed by values of FSC_n_ that are zero beyond 10 Å. The map shows 6.4 Å resolution. (c) The Xmipp package has been used with a single reference and “sub-optimal FSC” weighting to apply low-pass filtering at each cycle. With “sub-optimal” FSC weighting however, some overfitting and exaggerated resolution is seen. (d) Results of processing the same data using a new program still under development (McMullan, unpublished) and configured to show substantial overfitting when refined out to 5 Å. FSC_true_ (green symbols) shows the true resolution of the structure after removing the effect of overfitting on FSC_t_. (For interpretation of the references to colour in this figure legend, the reader is referred to the web version of this article.)

A data set was then collected at 300 keV using a backthinned Falcon CMOS direct electron detector. The result of processing 43758 particle images from 89 micrographs using Frealign is shown in Fig. 3(a). Overfitting when orientations are refined to 7 Å resolution is again observed but this was initially smaller than that observed with the film data at lower resolution. We conclude that low-resolution data is more readily overfitted; the ratio of parameters (3 orientations and 2 translations) to data (Fourier components from the structure that are above the noise level) is less favourable. To produce easily visible overfitting, we altered the automatic weighting scheme that Frealign normally uses to calculate correlation coefficients by using a value for the RB-factor of −200 Å^2^, effectively amplifying the contribution of the high resolution information to the correlation function (i.e. both the signal and the noise in the raw images). Using the recommended value of RB-factor (0 Å^2^) gave less overfitting, whereas a larger value (−300 Å^2^) gave more overfitting. Fig. 3(a) also shows how the FSC curves change when HR-noise substitution is carried out at different resolutions, in this case at 10 Å or 8.5 Å. The result of applying the formula (4) to deduce FSC_true_ from FSC_t_ and FSC_n_, is plotted in purple. This shows that the resolution of the final 3D map was 6.9 Å. The same resolution was obtained when the RB-factor used was 0 Å^2^. Since this resolution is slightly beyond the 7.0 Å resolution used for orientation refinement, it is unaffected by the HR-noise control. In the region between 7.0 Å and 10.0 Å resolution, the effect of overfitting is fairly small, but without carrying out the HR-noise control, we would not have known its extent. It is also clear that the quality of the 300 keV/Falcon experimental single particle images is significantly better than for those obtained at 80 keV on film.

The 300 keV data set was then processed using Relion [10] or Xmipp [53,54]. In this case, we compared the use of the “gold-standard” FSC weighting scheme that completely separates the data into two halves, advocated recently by Scheres [44] with the use of “sub-optimal” FSC weighting using a single common 3D reference. The gold-standard procedure is when the FSC between two independent halves of the data is used only to provide the weighting used in the parameter determination, whereas sub-optimal is when one 3D model is used for parameter determination using the FSC between the two halves of the data, which are no longer independent. The gold-standard procedure was done using Relion, and is shown in Fig. 3(b), whereas the sub-optimal procedure was done with Xmipp and is shown in Fig. 3(c). It is clear that the gold-standard FSC weighting procedure produces no overfitting, as expected since the two halves of the data have never been allowed to interact other than to produce the weighting, whereas the more conventional FSC weighting produces a much more significant degree of overfitting. The plots shown in Fig. 3(c) are fairly typical of what would be expected with other state-of-the-art image processing procedures.

The HR-noise substitution test is also valuable for program development. The procedure we describe can be used to improve the algorithm in any new procedure with the goal of minimising overfitting and maximising resolution. We have therefore tested it on a new, unpublished 3D reconstruction procedure in the process of being developed by one of us (GM), and the results are shown in Fig. 3(d). The new features of this program include the calculation of the 3D map after each cycle using a more sophisticated matrix inversion procedure and a computationally more efficient parameterisation. However, the susceptibility to overfitting and the ability to extract the best map from the data were unknown. For illustrative purposes only, we show the results of an early evaluation in which the amount of overfitting was particularly high. Clearly, the results shown are unacceptable in practice, but they do illustrate how valuable the HR-noise substitution test is. Obviously, the program will be improved and would not normally be used in the way shown here.

**Fig. 4:**
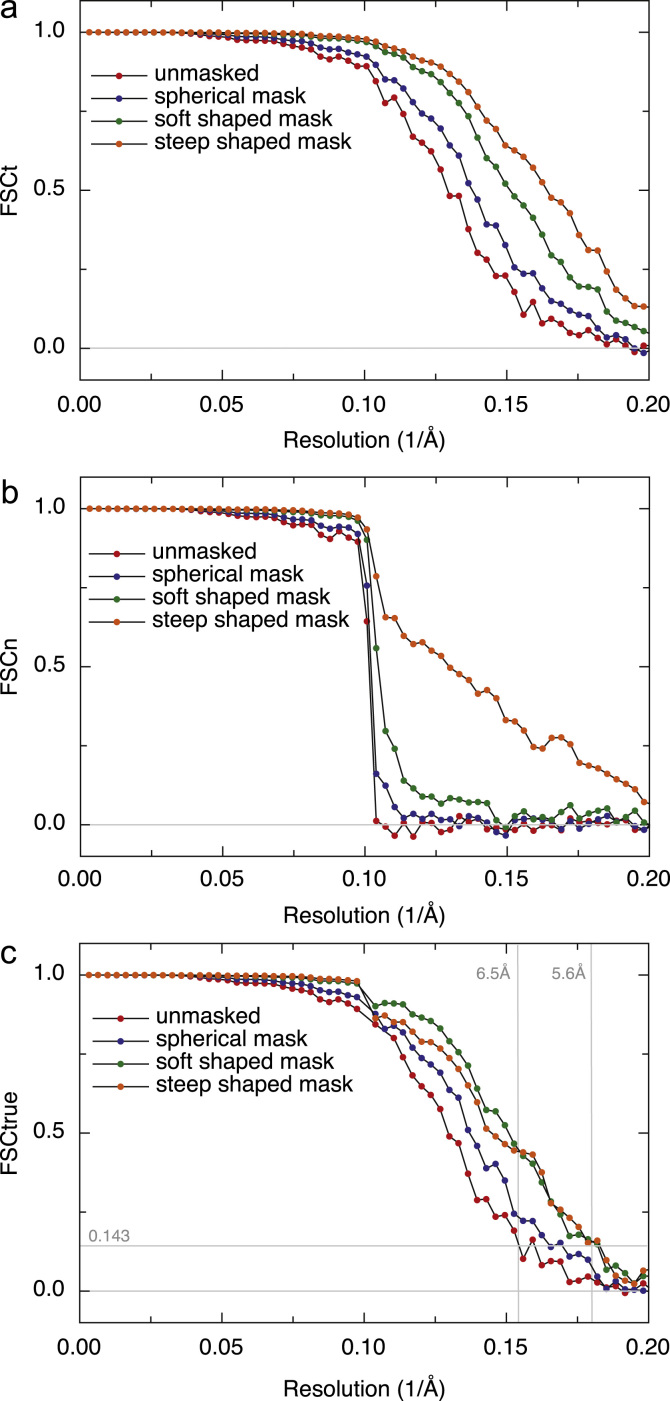
Effect of increasingly tight masking on FSC_t_ and FSC_n_, showing the usefulness of Eq. (4) in deconvoluting the effect of the mask to reveal the true FSC for the experimental density inside the mask (FSC_true_). (a) FSC_t_ curves between 3D maps from two halves of a data set. (b) FSC_n_ between 3D maps from the two halves of the HR-noise data set. (c) FSC_true_ curves calculated from the experimental plots shown in (a) and (b) using Eq. (4). The four curves were obtained by application of different masks to the same density map. The densities and shapes of features in the map are unaffected by masking. The red symbols are for unmasked maps. The blue symbols are for maps with a soft spherical mask slightly bigger than the molecule. The green symbols are for maps with a soft mask that follows the molecular shape. The mask fall-off profile for both soft masks was a cosine half-bell of width 6 pixels. The orange symbols are for maps with a steeper mask profile that follows the molecular shape, with a cosine half-bell of width 3 pixels. The FSC increases as background regions are excluded, with the resolution judged at 0.143 FSC increasing from 6.5 Å to 5.6 Å with an optimal mask. The tightest mask had a relatively steep profile that introduced false features in the FSC_t_ and FSC_n_ plots, but these are effectively removed by calculating FSC_true_, so that the orange and green FSC_true_ curves in (c) are very similar. An even steeper (e.g. binary) mask produces much larger artefacts in FSC and the deconvolution is then inaccurate. (For interpretation of the references to colour in this figure legend, the reader is referred to the web version of this article.)

Although the HR-noise substitution procedure was designed to show the effect of overfitting random noise in cryoEM images and its propagation into the final 3D map, we discovered that the HR-noise map can also be used to measure the effect of different types of mask on the FSC curves. The effect on FSC_t_ and FSC_n_ of 3D masking at three increasingly stringent levels is shown in Fig. 4 alongside the FSC curves from the unmasked maps. The genuine improvement in FSC as featureless regions are masked out is apparent, as well as the introduction of false artefacts in FSC if a tight mask with a very sharp boundary is used. Nevertheless, as long as the mask fall-off profile is not too steep, its artefactual effect on FSC_t_ and FSC_n_ is similar and the same Eq. (4) can be used to deconvolute real from false FSC correlations. The origin of masking artefacts on FSC curves is well known: multiplying any real-space density by application of a mask is equivalent to convolution of the transform of the original density with that of the mask. Thus, any sharp discontinuities in the mask introduce features in the transform that extend to higher resolution than any real information in the structure. This is seen clearly with the tightest mask in Fig. 4(a) and (b).

**Fig. 5:**
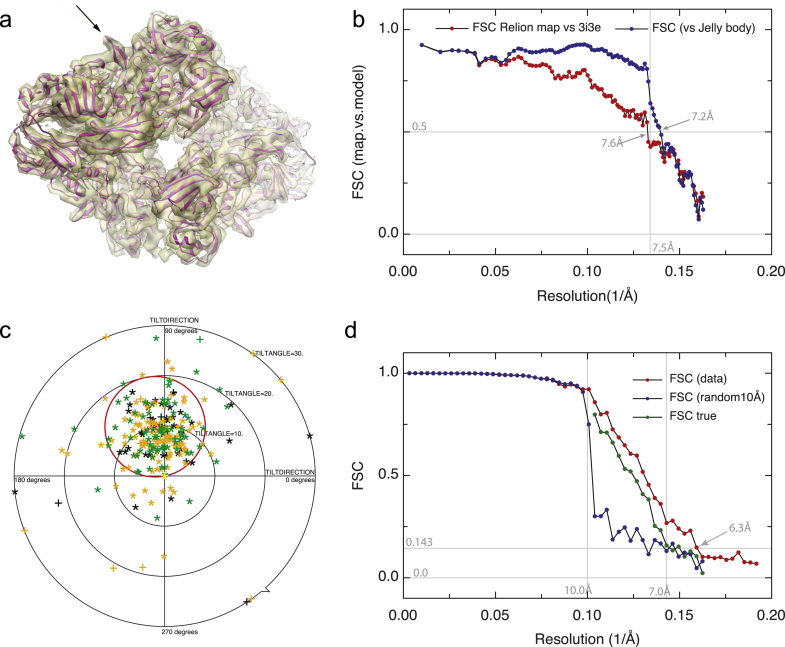
Summary of four recommended validation tools. Each panel is from a map chosen to show the value of each tool most clearly. (a) and (b) show comparisons of experimentally determined maps of beta-galactosidase with an atomic model, whereas (c) and (d) show tools that can be used with maps where atomic coordinates are not available. If atomic coordinates are available, then the FSC between map and model provides validation of all steps in the process, although great care is still needed with any flexible fitting procedure. In many cases, however, such as for novel structures at low resolution, atomic coordinates are not available. (a) 3D map of beta-galactosidase from Fig.3(b) obtained using Relion [10] with rigid-body-fitted atomic model superimposed. The arrow shows a beta hairpin. (b) FSC between the Relion map and atomic model. A resolution of 7.6 Å is estimated at 0.5 FSC for a rigid body fit, and 7.2 Å using a jelly body flexible fit to data truncated at 7.5 Å. The value of FSC 0.5 is used in this comparison because the map is calculated from all the images rather only half and the atomic model is assumed to be perfect. (c) Tilt pair parameter plot, which is important for validation at lower (below 1/15 Å^−1^) resolutions [27]. (d) A typical comparison of the FSC curve (from Fig.3(c)) for a structure with that obtained after HR-noise substitution. In this case, the correlation between the two half data sets shows that about one third of it consists of overfitted noise at 9 Å and half at 7 Å. A genuine resolution of 7.0 Å is estimated from FSC_true_ (green symbols), rather than the 6.3 Å value that would be falsely suggested by the overfitted noise (red symbols). The value of FSC 0.143 is used in this comparison because both maps are calculated from only half the images [27] so both contain more noise than the map used for (b). (For interpretation of the references to colour in this figure legend, the reader is referred to the web version of this article.)

Lastly, in Fig. 5, we show a collage of validation tools. We have juxtaposed the new HR-noise substitution procedure we describe in this paper (Fig. 5(d)) with three other tools that are available to allow the experimenter to test the validity of a single particle structure.

Fig. 5(a) shows a conventional superposition of map and model. It is clear that features such as helices can be resolved but that the individual strands of the beta-sheets cannot, which would need higher resolution. At top left, a beta-hairpin (see arrow) is clearly visible.

Fig. 5(b) shows the FSC between the Relion map and two models. One of the models is a simple rigid body fit of the coordinates 3I3E [49], showing a resolution of 7.6 Å at FSC 0.5. The other model has been flexibly fitted using a reciprocal space procedure called jelly body fitting [55] using data with a cut-off at 7.5 Å. It shows a slight improvement in the FSC 0.5 resolution, from 7.6 Å to 7.2 Å. Note that if flexible fitting or jelly body fitting is carried out using higher resolution data, the same kind of overfitting of the model to the map that we have been trying to minimise in the map calculation will occur. More work is still needed to decide how to validate the fitting or docking of models into cryoEM maps; perhaps limiting the resolution of the fitting or docking to below the known, validated map resolution would be appropriate. An increase in the FSC beyond the cut-off resolution used might then indicate that the resulting flexibly-fitted model is an improvement over the rigid body docking. In this case, the improvement is slight.

The best resolution between map and flexibly-fitted model shown in Fig. 5(b) is 7.2 Å at FSC 0.5, rather than the 6.4 Å gold-standard resolution at FSC 0.143 for the unmasked 3D map shown in Fig. 3(b). It would be expected that these two resolution estimates would be the same if the model were perfect [27]. A comparison of the map with the model indeed shows some new features (to be described elsewhere) that are missing from the X-ray structure, supporting the conclusion that the model can be improved.

Fig. 5(c) shows the application of the tilt pair parameter plot (TPPP) procedure [27] to beta-galactosidase images recorded under the same conditions as used for the main 300 keV Falcon data set. The clustering around the expected tilt axis and tilt angle used to collect the tilt pair images is sharper than obtained previously from 80 keV CCD data [26], and provides convincing validation of the quality of the images and the 3D model of beta-galactosidase.

Finally, Fig. 5(d) shows how application of Eq. (4) to the FSC_t_ and FSC_n_ curves shown in Fig. 3(c) changes the estimated resolution from 6.3 Å to 7.0 Å.

## 4 Discussion

There is some similarity between our proposed resolution and overfitting test and the use of the free *R*-factor in X-ray crystallography [56]. In the X-ray case, a small amount of diffraction data (5%) is set aside and becomes the test data and the bulk of the data (95%) are used in refinement. As refinement progresses, the value of *R*_free_ and *R*_work_ are both calculated. *R*_work_ always decreases since it refers to the data being used in refinement, but *R*_free_ decreases only if the model is genuinely improving and by a greater amount when the improvement is greater. The difference between *R*_free_ and *R*_work_ measures the amount of overfitting, whereas the value of *R*_free_ gives an unbiased estimate of how well the model agrees with the observations as a function of resolution. In our case, no data are excluded from the structure determination; in this sense it differs from the free *R*-factor approach. Instead, in the parallel set of HR-noise substituted data, noise replaces the data beyond a resolution where the real structural information is sufficiently small in total power (estimated to be less than 5%) that it has a negligible effect on the accuracy of orientation determination in the HR-noise control data set. Any non-zero value of the FSC_n_ above the resolution cut-off used in the HR-noise test gives a measure of the amount of overfitting, whereas the difference between the two curves (FSC_t_ and FSC_n_) gives an unbiased estimation of resolution. As we have shown, it is also possible to deconvolute the unbiased part of the FSC_t_ curve using FSC_n_ to give FSC_true_.

In the X-ray case, overfitting becomes apparent as a wider gap between *R*_free_ and *R*_work_, for example more than 5%. Resolution is estimated from the point at which *R*_free_ exceeds a threshold value that is agreed by the community based on experience, normally around 30-35%. In cryoEM, there is no consensus yet on an acceptable amount of overfitting and what threshold value of FSC should be used in resolution estimation. We would argue that it is simplest to use one of the established procedures [e.g. 10] to obtain a map with no overfitting and to determine resolution using the 0.143 criterion. However, clearly a small proportion of overfitting of, for example, less than one third of the total power in a particular resolution shell simply introduces some extra noise into the 3D map that can be dealt with by using less sharpening or more damping. Consequently, we argue that any method of cryoEM structure analysis can be used even when it is known to be susceptible to overfitting, provided it is accompanied by the HR-noise test. The HR-noise test is also compatible with the proposed introduction of single particle Wiener filters that restrict the FSC to the region of the 3D map occupied by the particle [57,58]. Any false correlations introduced into FSC_t_ by the mask will also appear in FSC_n_, thus maintaining the integrity of the HR-noise test, as shown in Fig. 4.

Note that the appearance of a large degree of overfitting, as seen for example in Fig. 3(d), is not necessarily always bad. Since the power of the high resolution Fourier components in the region of overfitting may be quite weak, its presence may not change the interpretability of the map at the lower resolution. However, knowledge of the extent of overfitting makes the experimenter aware of the problem and prevents over-optimism about map resolution. The derived function FSC_true_ would then be the correct one to use in calculating signal-to-noise weighting of the map using the Cref formula, sometimes called figure-of-merit (FOM) weighting, proposed by Rosenthal and Henderson [27]. In Fig. 3(d), for example, calculation of FSC_true_ gives an reasonable resolution estimate of 6.5 Å and because the power at high resolution was very weak, the map was not unduly noisy.

Note also that the slightly different resolutions obtained using the different programme packages after application of Eq. (4), shown in Figs. 3 and 5 ranging from 6.4 Å to 7.0 Å, are likely to arise for reasons unconnected with the central point of this paper. For example, different approaches to masking and more-or-less sophisticated models for the resolution-dependence of the signal and noise, will affect the final map quality. The HR-noise substitution approach successfully measures the amount of overfitting and derives a resolution estimate that is free of overfitting in every situation. This can then be used to decide how to optimise use of a particular package for a particular project.

Stewart and Grigorieff used a related approach to the one we have introduced here when exploring noise bias. In their 2003 paper [28], they left out a zone of reciprocal space data from their phantom but retained the same noise distribution. They used this to find the weighting function that would best minimise overfitting as judged by the magnitude of the FSC in the omitted zone. The weighting scheme was then implemented in Frealign. They used model calculations with noise but no signal to measure overfitting in Frealign, whereas we suggest that high-resolution noise substitution can be used to measure overfitting with real images using any package or combination of packages, as well as for program development.

There is one important parameter in the HR-noise test: the resolution beyond which HR-noise should be introduced. If it is too low, then the particle orientations will be degraded in the HR-noise comparison and the difference between the two FSC curves will give a false reassurance about resolution. If the resolution is too high, then all the HR-noise will be beyond the resolution where the FSC becomes weak, and the exercise will be uninformative. It is therefore important to choose a resolution where the signal from the structure is sufficiently weak that it contributes little or nothing to the accuracy of the orientation determinations, yet is strong enough to be distinguished from noise in the 3D map. The tilt pair approach showed that most of the information which determines orientation in current images is at low or medium resolution, below 15 Å. Scheres [10,41] introduced an equation that allowed him to estimate the contributions of different spatial frequencies to the orientation determination, showing very little contribution at high resolution. For the beta-galactosidase data in Fig. 3(a), the FSC falls to 87% at 10 Å, 75% at 9.2 Å and 50% at 8.2 Å. It is clear therefore that any choice between 8.5 Å and 10 Å would be satisfactory. In most of this paper, we have used a 10 Å threshold for the HR-noise substitution. In future, progress with cryoEM technology or new methods of specimen preparation will provide improved images, so the chosen HR-noise resolution can obviously be extended to higher resolutions as cryoEM resolutions improve. A suitable choice might be the resolution where FSC_t_ falls to 75 or 80%.

The procedure we describe represents a new tool that can be used alongside others to help the experimenter to judge the quality of a 3D single particle structure. It can also measure the robustness of an existing or new protocol in protecting against any intrinsic tendency to overfitting. We hope it will be generally useful both for programmers and experimentalists.

## 5 Conclusions

In single particle electron cryomicroscopy, there are two accepted ways to prevent overfitting. One way is to compare maps obtained by dividing the data in half, and processing each set in a completely independent way throughout the refinement; the resolution obtained by comparing how well the two maps agree is then used to filter a map obtained using all the data. The other way is to limit the resolution for determination of orientation parameters. Some individuals may prefer other methods that have some susceptibility to overfitting, but may not know whether their resulting structure is compromised. The method of HR-noise substitution described in this paper can measure overfitting and validate the map resolution with any image processing procedure. It is also useful in deconvoluting the effect of applying a molecular envelope or mask.

## 6 Declaration of contribution

Chen prepared cryo-specimens and carried out the electron microscopy data collection. Faruqi and McMullan helped to develop the detector used in collecting the 300 keV images. Scheres performed computer analysis using the Relion and Xmipp software packages, used in Fig. 3b and c respectively. Short performed a similar computer analysis using the Spider software package that is not included. McMullan performed computer analysis using his unpublished 3D single particle structure analysis package, used in Fig. 3d. Henderson and Scheres performed the tests of the effect of masking shown in Fig. 4. Murshudov carried out the jelly-body flexible fitting used in Fig. 5b. Henderson performed computer analysis using Frealign, used in Fig. 2 and Fig. 3a, and coordinated the overall plan for the paper. All authors have read and agreed the text.
